# Antimicrobial Peptides Human Beta-Defensin-2 and -3 Protect the Gut During *Candida albicans* Infections Enhancing the Intestinal Barrier Integrity: *In Vitro* Study

**DOI:** 10.3389/fcimb.2021.666900

**Published:** 2021-06-10

**Authors:** Alessandra Fusco, Vittoria Savio, Maria Donniacuo, Brunella Perfetto, Giovanna Donnarumma

**Affiliations:** Department of Experimental Medicine, University of Campania “Luigi Vanvitelli”, Naples, Italy

**Keywords:** *Candida albicans*, microbiota, antimicrobial peptides, gut, tight junctions

## Abstract

The intestinal mucosa is composed of a monolayer of epithelial cells, which is highly polarized and firmly united to each other thanks to the presence of proteins complexes, called Tight junctions (TJs). Alteration of the mucus layer and TJs causes an increase in intestinal permeability, which can lead to a microbial translocation and systemic disorders. *Candida albicans*, in addition to its role of commensal, is an opportunistic pathogen responsible for disseminated candidiasis, especially in immunocompromised subjects where the dysbiosis leads to damage of the intestinal mucosal barrier . In this work, we used a line of intestinal epithelial cells able to stably express the genes that encodes human beta defensin-2 (HBD-2) and -3 (HBD-3) to monitor the invasion of *C. albicans in vitro.* Defensins are a group of antimicrobial peptides (AMPs) found in different living organisms, and are involved in the first line of defense in the innate immune response against pathogens. The results obtained show that the presence of antimicrobial peptides improves the expression of TJs and increases the Trans Epithelial Electrical Resistence value. In addition, the invasive ability of *C. albicans* in transfected cells is significantly reduced, as well as the expression levels of genes involved in the apoptotic pathway. Through the study of interaction between antimicrobial peptides and microbiota we will be able in the future to better understand the mechanisms by which they exert the host defense function against intestinal pathogens.

## Introduction

The human intestinal microbiota is a complex ecosystem composed mainly of bacterial cells but also archaea, viruses and eukariota such as parasites and fungi. Its composition is unique and peculiar to each individual and differs along the digestive tract, according to the environmental influences and lifestyle stimuli. Additionally, it sustains gut integrity and prevents gut colonization by pathogens (Kataoka, 2016; Partida-Rodríguez et al., 2017; Sommer et al., 2017). Among the intestinal microbiota, of great interest is the *Candida albicans* species. It is an opportunist pathogen belong to the family of *Saccharomycetaceae*, which can grow in a variety of morphological forms such as yeast, pseudohyphae and hyphae (Sudbery et al., 2004; Nadeem et al., 2013), present on the mucosal surfaces of most healthy individuals and which can cause superficial to severe systemic infections mostly originating from the gastrointestinal tract (Paoletti et al., 2013; Höfs et al., 2016; Bertolini et al., 2019), especially in immune-compromised patients. The major risk factor for disseminated candidiasis includes damage to the mucosal intestinal barrier and dysbiosis with alteration of the resident microbiota (Allert et al., 2018).

Invasion by *C. albicans* also actively contributes to enterocyte damage with consequent cell death (Goyer et al., 2016; Allert et al., 2018) probably due to the activation of mechanisms involving necrosis and/or apoptosis (Villar and Zhao, 2010; Villar et al., 2012; Wagener et al., 2012; Moyes et al., 2015; Goyer et al., 2016).

The integrity of the intestinal barrier is fundamental for gut health and homeostasis; this condition is maintained by a mucus layer of epithelial cells held together by a complex system of intercellular junctions including weak junctions (i.e. Adherens and Gap junctions, Desmosomes) and apical protein complexes (Oshima and Miwa, 2016; Sturgeon and Fasano, 2016; Capaldo et al., 2017; Tsukita et al., 2019) called “tight junctions” (TJs). Adherens Junctions (AJ), mainly composed of proteins of the cadherin family, are located immediately below TJ while Desmosomes and Gap Junctions are distributed on the lateral side of the enterocyte (Schneeberger and Lynch, 2004). TJs are represented by several multiprotein complexes that include transmembrane proteins, such as Claudin and Occludin, Junctional Adhesion Molecules (JAM) and others, interacting between themselves (both hemophilic and heterophilic interactions) and with intracellular scaffolding proteins anchored to the actin cytoskeleton, including Zonula Occludens (ZOs) (Schneeberger and Lynch, 2004; Oshima and Miwa, 2016; Sturgeon and Fasano, 2016; Capaldo et al., 2017; Tsukita et al., 2019). These interactions preserve the integrity of the tight junction and regulate the passage of solutes, water, large molecules and ions through the paracellular space.

The mucus layer consists instead of globet cells secreting glycoproteins called mucins which act as a barrier at the host-lumen interface. Moreover, many bioactive metabolites produced by intestinal epithelial cells accumulate in the mucous layer in order to enhance the barrier function by influencing the composition of the gut microbiota. Among these components, there are antimicrobial peptides (AMPs). As important components of the animal innate immune system, AMPs have a strong immunomodulatory and antimicrobial properties and are able to activate and modulate the adaptive immune responses (Lai and Gallo, 2009; Donnarumma et al., 2016; Liang and Diana, 2020; Ma et al., 2020). Recent studies demonstrated that AMPs are involved in the regulation of mucin and TJs expression and microbiota composition (Liévin-Le Moal and Servin, 2006; Muniz et al., 2012; Kiatsurayanon et al., 2014). In particular, human beta-defensin-2 and -3 (HBD-2 and HBD-3) exhibit a wide variety of immunomodulatory functions, production of chemokines and cytokines, cell proliferation, suppression of pro-inflammatory responses, and promote wound healing (Otte et al., 2008; Fusco et al., 2017; D’Agostino et al., 2019). It was widely demonstrated that MUC-2 expression is enhanced in human epithelial Caco-2 cells in response to HBD-2 (Otte et al., 2008; Cobo et al., 2015), and, in turn, upregulation of MUC-2 promotes HBD-2 expression (Cobo et al., 2015; Takiishi et al., 2017), suggesting a mutual positive regulating mechanism between HBD-2 and MUC-2. HBD-3 has been shown to trigger the synthesis of multiple TJ proteins (Purchiaroni et al., 2013; Takiishi et al., 2017).

The alteration of the mucus layer and TJs results therefore in an increase of intestinal permeability, which can lead to an increase in bacterial translocation, inflammatory state and consequent systemic disorders (Oshima and Miwa, 2016; Sturgeon and Fasano, 2016; Capaldo et al., 2017; Tsukita et al., 2019).

Recent studies (Allert et al., 2018; Basmaciyan et al., 2019) show that the TJs, in conditions of barrier integrity, represent an obstacle to the penetration of *C. albicans* inside the enterocytes in the early stages of interaction between the fungus and the intestinal epithelium. On the other hand, it is known that fungi can traverse epithelial cell barriers by proteolytic degradation of intercellular tight junctions (Sheppard and Filler, 2014).

Recently, we created a line of intestinal epithelial cells able to stably express genes encoding human HBD-2 and HBD-3 (Fusco et al., 2017) to test their role in *Salmonella typhimurium* infections. Interestingly, it was found that both HBD-2 and HBD-3 are able to significantly reduce the inflammatory state of cells infected with *Salmonella*, but they are also able to enhance the activity of probiotic strains in counteracting the pathogenesis of infections sustained by this pathogen. These data encouraged us to pursue further investigations in order to better elucidate the relationships between antimicrobial peptides and gut microbiota microorganisms.

In this work, with the aim to monitor *C. albicans* invasion due to the host cell damage, Caco-2 cells untransfected and the HBD2/HBD3-transfected were used in an experimental model of *C. albicans* invasion *in vitro*. The loss of monolayer integrity in both experimental cells conditions has been evaluated via quantification of trans epithelial electrical resistance (TEER) and through the molecular and protein evaluation of the target proteins of barrier damage.

## Materials and Methods

### Cloning and Transfection

Total RNA was extracted using a High Pure RNA Isolation Kit (Roche Diagnostics) from primary cultures of human keratinocytes stimulated with the LPS of *Pseudomonas aeruginosa* and TNF-α to obtain a high production of antimicrobial peptides. It was subsequently transcribed into complementary cDNA using random hexamer primers (Random hexamers, Roche) at 42°C for 45 minutes, according to the manufacturer’s instructions. Two pairs of degenerate primers, designed on their specific amino acid sequence (**Table 1**) were used to amplify, by Real-Time PCR, genes coding HBD-2 and HBD-3 with FastStart High Fidelity (Roche Diagnostics).

**Table 1:** Primer sequences and amplification programs.

Gene	Primers sequence	Conditions	Product size (bp)	Ref
HBD-2	5’-CCAGCCATCAGCCATGAGGGT-3’ 5’- GGAGCCCTTTCTGAATCCGCA-3’	10’’at 95°C, 5”at 60°C, 10” for 40 cycles	254	Fusco et al. (2017)
HBD-3	5’- CGGCAGCATTTTGCGCCA-3’ 5’- CTAGCAGCTATGAGGATC-3’	10’’at 95°C, 4”at 58°C, 8” for 40 cycles	206	Fusco et al. (2017)
Occludin	5’-TCAGGGAATATCCACCTATCACTTCAG-3’ 5’-CATCAGCAGCAGCCATGTACTCTTCAC-3’	10’’at 95°C, 45”at 60°C for 40 cycles	188	Cuffaro et al. (2020)
Zonulin-1	5’-AGGGGCAGTGGTGGTTTTCTGTTCTTTC-3’ 5’-GCAGAGGTCAAAGTTCAAGGCTCAAGAGG-3’	10’’at 95°C, 45”at 60°C for 40 cycles	217	Wilms et al. (2019)
Claudin-1	5’-CTGGGAGGTGCCCTACTTTG-3’ 5’-ACACGTAGTCTTTCCCGCTG-3’	1’’at 95°C, 30” at 60°C, 20’’at 72°C for 40 cycles	128	Wu et al. (2018)
MUC-2	5’-CTGCACCAAGACCGTCCTCATG-3’ 5’-GCAAGGACTGAACAAAGACTCAGAC-3’	5’’at 96°C, 16” at 60°C, 8’’at 72°C for 40 cycles	401	Perrais et al. (2002)
Bcl-2	5’-CAGCTGCACCTGACGCCCTT-3’ 5’-CCCAGCCTCCGTTATCCTGGA-3’	5’’at 94°C, 7” at 58°C, 9’’at 72°C for 40 cycles	235	Fusco et al. (2018)
Fas-L	5’-GGATTGGGCCTGGGGATGTTTCA-3’ 5’-TTGTGGCTCAGGGGCAGGTTGTTG-3’	5’’at 95°C, 7” at 60°C, 14’’at 72°C for 40 cycles	344	Fusco et al. (2018)
Fas-R	5’- CCAAGTGACTGACATCAACTC-3’ 5’- CTCTTTGCACTTGGTGTTGCTGG -3’	5”at 94°C, 8”at 55°C, 17”at 72°C for 40 cycles	426	Fusco et al. (2018)
β-actin	5’-GACGACGACAAGATAGCCTAGCAGCTATGAGGATC-3’ 5’-GAGGAGAAGCCCGGTTAACTTCCGCAGCATTTTGCGCCA-3’		243	Fusco et al. (2018)

The amplified DNA fragments were subjected to restriction and sequencing analysis and cloned into the pEF/V5-HIS TOPO (Invitrogen) vector using the T4 DNA ligase (Invitrogen), in accordance with the manufacturer’s protocol, and then transformed into *Escherichia coli* TOP 10 (Invitrogen).

The cloning vectors, pEF/V5-HIS TOPO-HBD2 and pEF/V5-HIS TOPO-HBD-3, were extracted from the bacterial culture and amplified using a QIAprep Spin Midiprep Kit (QIAGEN).

Caco-2 cells were transfected using the IBAfect reagent (IBA), according to the manufacturer’s manuals. Briefly, 3 x 10^5^ cells were seeded in 6-well plates, and immediately after seeding, plasmids conjugated with the transfection reagent were added. The mixture was incubated for 24 and 48 hours. After incubation, the success of the experiment was verified by the extraction of mRNA from treated cells and by the amplification of HBD-2 and HBD-3 genes by PCR.

Cell-free supernatants of the transfected cells were recovered by centrifugation and assayed for the HBD-2 and HBD-3 concentration by an enzyme-linked immunosorbent assay (Elabscience and Abcam, respectively).

For blasticidin selection, untransfected and transfected cells were cultured at 37°C and 5% CO_2_ for 14 days in the presence of the following increasing concentrations of blasticidin S (Sigma-Aldrich): 5, 10, 20, 50, 100 and 250 μg/ml. Then, MTT labeling reagent was added at a final concentration of 0.5 mg/ml. After 4 hours, a solubilization solution was added to each well and the plates were incubated overnight. Spectrophotometric absorbance was measured using a microplate (ELISA) reader at a wavelength of 570 nm.

### Cell Culture

Caco-2 cells (Human Caucasian colon adenocarcinoma cells, ATCC^®^ HTB-37™) were routinely cultured in Dulbecco’s modified Eagle medium (DMEM, Gibco) supplemented with 1% Penstrep, 1% glutamine and 10% fetal calf serum (Microgem) at 37°C at 5% CO**_2_**. After transfection, as described in our previous work (Fusco et al., 2017), the cells were grown in the presence of 250 μg/ml Blasticidin (InvivoGen) in a sterile 25 cm^2^ flask at a concentration of 3 x 10^5^ to confluence for 21 days to reach full differentiation and polarization (see **Supplementary Materials**). The culture medium was changed every two days.

### Evaluation of TJs, MUC and AMPs Genes

To evaluate *Occludin*, *Zonulin-1*, *Claudin-1, MUC-2*, *HBD-2* and *HBD-3* gene expression, Caco-2 cells, cultured as described above, were used for total RNA extraction with High Pure RNA Isolation Kit (Roche; Milan, Italy). Two hundred nanograms of total cellular RNA were reverse-transcribed (Expand Reverse Transcriptase, Roche; Milan, Italy) into complementary DNA (cDNA) using random hexamer primers (Random hexamers, Roche; Milan, Italy) at 42°C for 45 minutes, according to the manufacturer’s instructions.

Real-time PCR was carried out with the LC Fast Start DNA Master SYBR Green kit using 2 µL of cDNA, corresponding to 10 ng of total RNA in a 20 μL final volume, 3mM MgCl2 and 0.5 μM sense and antisense primers (**Table 1**). After amplification, the melting curve analysis was performed by heating the sample to 95°C for 15 seconds with a temperature transition rate of 20°C/s, cooling to 60°C for 15 seconds with a temperature transition rate of 20°C/s, and then heating the sample at 0.1°C/s to 95°C. The results were then analyzed using LightCycler^®^ software (Roche Diagnostics). The standard curve of each primer pair was established with serial dilutions of cDNA. All PCR reactions were run in triplicate. The specificity of the amplification products was verified by electrophoresis on a 2% agarose gel and visualization by ethidium bromide staining (Fusco et al., 2017).

### Western Blot Analysis

After treatment, the cells were scraped with 1 ml of PBS, and the cell pellet was homogenized with 300 ml of ice-cold buffer [150mM NaCl, 1%, 10mMTris-HCl pH 8, EDTA 2mM, Triton X 100 4, EGTA 2mM] supplemented with 1mM phenylmethylsulfonyl fluoride (PMSF), 1mM sodium orthovanadate (Na_3_VO_4_) and 50mM NaF. Total extracts were cleared by centrifugation for 30 minutes at 4°C at 10,000 x g and assayed for protein content by Bradford’s method. Fifty micrograms of protein from each cell lysate were separated by a 7% sodium dodecyl sulphate-polyacrylamide gel electrophoresis (SDS-PAGE) and transferred to nitrocellulose membranes before staining the filters with 10% Ponceau S solution for 2 minutes to verify equal loading and transfer efficiency (Paoletti et al., 2013). Blots were blocked overnight with 5% non-fat dry milk and then incubated with anti-ZO-1, anti-Claudin (Bethyl Laboratoires.Inc, Montgomery, TX) and anti-Occludin (Novus Biological, USA) antibodies, according to the manufacturer’s instructions, in Tris-buffered saline (TBS) (150mM NaCl and 20mM Tris–HCl, pH8) for 2 hours at room temperature. After washing with 0.1% Tween-20 PBS, the filters were incubated with 1:2000 peroxidase-conjugated immunoglobulins for 1 hour at room temperature. They were thoroughly washed and then analyzed using the ECL system (Amersham, Milan, Italy).

### Transepithelial Electrical Resistance (TEER) Measurement

TEER was used as a measure of cell monolayer integrity and differentiation. Caco-2, Caco-2/HBD-2 and Caco-2/HBD-3 were seeded and cultured in 24-well Transwell^®^ chambers (0, 4 μm pore size, Corning, NY,USA) at a density of 1x 10^5^ cells/cm^2^, and the medium was replaced every other day until 21 days. TEER was measured using EVOM2 Epithelial Voltohmmeter (WPI). The electrode was immersed at a 90° angle with one tip in the basolateral chamber and the other in the apical chamber. Care was taken to avoid electrode contact with the monolayer, and triplicate measurements were recorded for each monolayer. An insert without cells was used as a blank, and its mean resistance was subtracted from all samples. Unit area resistance was then calculated by dividing resistance values by the effective membrane area (0.33 cm^2^).

### *Candida albicans* Adhesion and Internalization Assay

A strain of *C. albicans* ATCC^®^ 6538 was cultured in Sabouraud dextrose agar medium (SAB-Oxoid). On the day of experiment, semiconfluent monolayer of Caco-2, Caco-2/HBD-2 and Caco-2/HBD-3 were infected with *Candida* at an exponential growth phase at a ratio of 1:10 cell/yeast for 2 hours. After this time, for adhesion assay, the cells were lysed by adding 1 ml of cold 0.1% Triton-X100 and plated in serial dilutions on Sabouraud Dextrose Agar (SAB-Oxoid) for 24 hours at 30°C to identify the viable intracellular yeast by counting the CFU/ml. For invasiveness assay, after the first 2 hours of incubation, the cell supernatants containing yeasts was removed and replaced with fresh medium containing nystatin (Sigma) at a mycocidal concentration (1.6 μg/ml) for additional 2 hours at 37°C, then the cells were lysed and CFU/ml was counted as described above (Baroni et al., 2018).

### *Candida albicans* Invasion Through Barrier Assay

The Caco-2, Caco-2/HBD-2 and Caco-2/HBD-3 cells were cultured for 21 days in 24-well Transwell^®^ chambers (8 μm pore size, Corning, NY,USA) at a density of 1x 10^5^ cells/cm^2^, and the medium was replaced every other day. At the end of this time, cells were infected with *C. albicans*, cultured as described above, at a concentration of 0,5 O.D., corresponding approximatively at 10^8^ CFUs/ml for 4 hours, then, with the aim to identify the viable yeast able to cross the barrier, the medium present in the basolateral side was plated in serial dilutions on Sabouraud Dextrose Agar (SAB-Oxoid) for 24 hours at 30°C by counting the CFUs/ml.

### Infection and Cell Damage Evaluation

Semiconfluent monolayer of Caco-2, Caco-2/HBD-2 and Caco-2/HBD-3 were infected with *C. albicans* at an exponential growth phase at a ratio of 1:10 cell/yeast for 6 and 24 hours. At the end of the first 6 hours, mRNA extraction and Real-Time PCR were carried out as described above in order to evaluate the expression of apoptotic genes *BCl-2*, *FAS-L* and *FAS-R* (**Table 1**) and of *HBD-2* and *HBD-3*. At the end of 24 h, resazurin was added to the cells at a concentration of 0,5 mg/ml and incubated for 4 hours to obtain percentage values of viable cells.

### ELISA Assay

Caco-2 cell monolayers were infected with *C. albicans* as described above for 24 hours. At the end of this time, supernatants were harvested, and the presence of MUC-2, Bcl-2 and FAS-L was analyzed by enzyme-linked immunosorbent assay (ELISA; Elabscience, US, ThermoFisher). In addition, ELISA assays for HBD-2 (Elabscience) and HBD-3 (Abcam) were performed on cell supernatants to verify that the overexpression of antimicrobial peptides in transfected cells remained constant during differentiation time and after infection with *C. albicans.*

### Statistical Analysis

Significant differences among groups were assessed through two-way ANOVA by using GraphPad. Prism 6.0, and the comparison between the means was calculated by t-student test. The data are expressed as means ± standard deviation (SD) of three independent experiments.

## Results

### Modulation of Defensins, TJs and Mucin Genes

**Figure 1:**
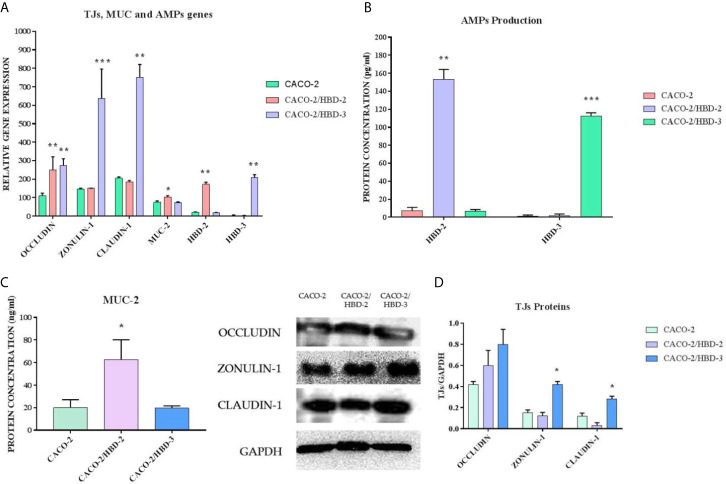
**(A)** Real-Time PCR results show the expression levels of AMPs and of intestinal barrier genes in Caco-2, Caco-2/HBD-2 and Caco-2/HBD-3, as relative gene expression. **(B)** ELISA assay for HBD-2 and HBD-3 production in Caco-2, Caco-2/HBD-2 and Caco-2/HBD-3. **(C)** ELISA assay for MUC-2 production in Caco-2, Caco-2/HBD-2 and Caco-2/HBD-3. **(D)** Western blot analysis for TJ proteins content in Caco-2, Caco-2/HBD-2 and Caco-2/HBD-3 cells. GAPDH was used as internal control of protein load. Data are representative of three different experiments ± SD. Significant differences are indicated by *p < 0.05, **p < 0.01, ***p < 0.001.

Caco-2, Caco-2/HBD-2 and Caco-2/HBD-3 cells were cultured for 21 days to achieve full differentiation. At the end of this period, the degree of expression of the genes encoding *Occludin*, *Zonulin-1*, *Claudin-1*, *MUC-2, HBD-2* and *HBD-3*, and the production of the corresponding proteins were analyzed. The results obtained (**Figure 1**) show that overexpression of defensin genes is preserved during the 3 weeks of differentiation, and that both antimicrobial peptides strengthen the integrity of the barrier, but in two different ways: HBD-2 significantly upregulates the expression of MUC-2 and strongly of Occludin but has no effect on the expression of the other TJs. HBD-3, on the other hand, although not upregulating the expression of MUC-2, induces a very strong increase in the expression of all the barrier proteins analyzed.

### Evaluation of Intestinal Permeability *In Vitro*

**Figure 2:**
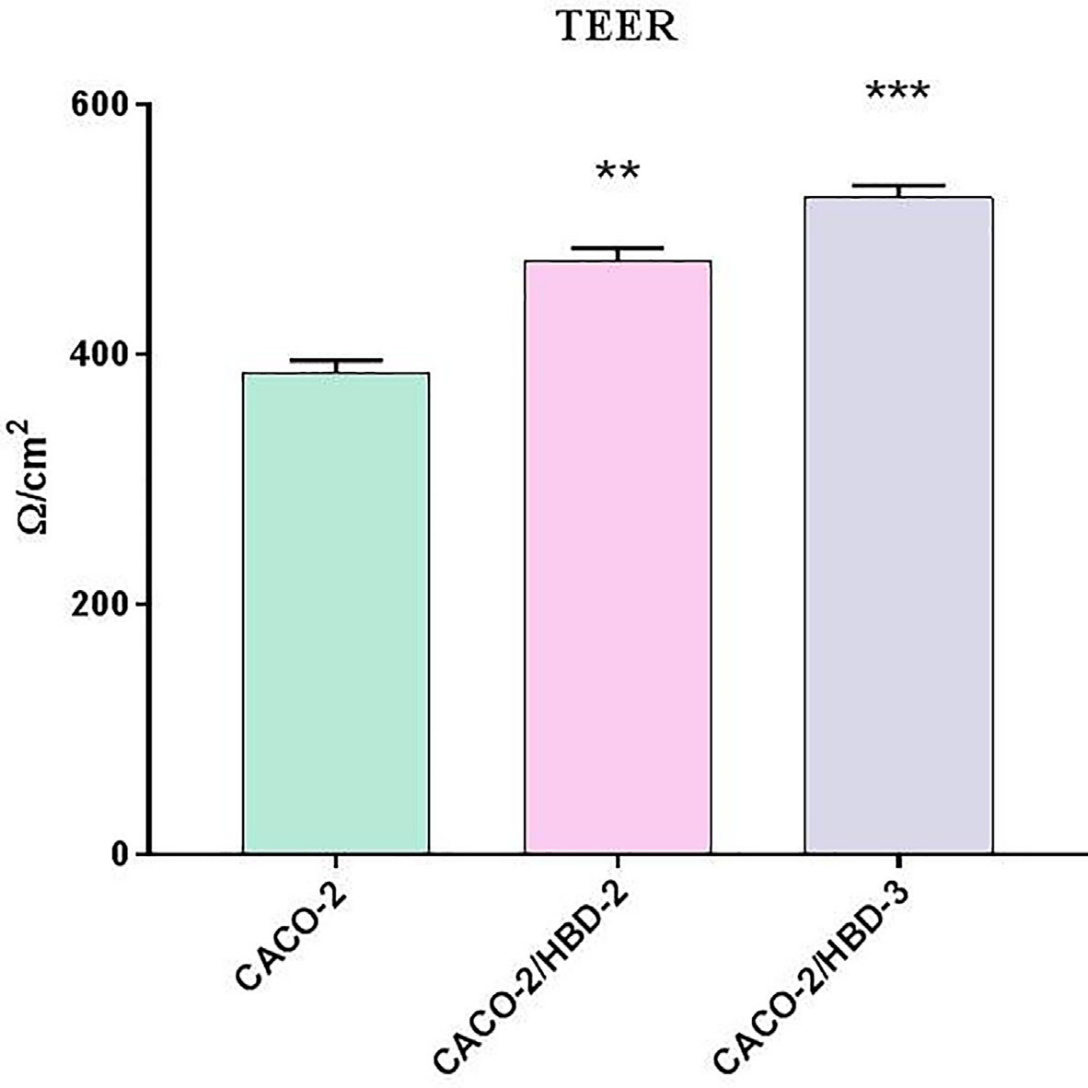
TEER measurement after 23 days of differentiation of transfected and untransfected Caco-2 cells. Data are representative of three different experiments ± SD. Significant differences are indicated by *p < 0.05, **p < 0.01, ***p < 0.001.

TEER is an indicator of epithelial paracellular permeability to ionic solutes and is used to evaluate intestinal barrier function. Compared with the Caco-2 cells, TEER consistently increased in the Caco-2/HBD-2 and Caco-2/HBD-3 (**Figure 2**), indicating that the presence of antimicrobial peptides could attenuate intestinal epithelial barrier dysfunction during pathological conditions.

### *C. albicans* Adhesion and Internalization

**Figure 3:**
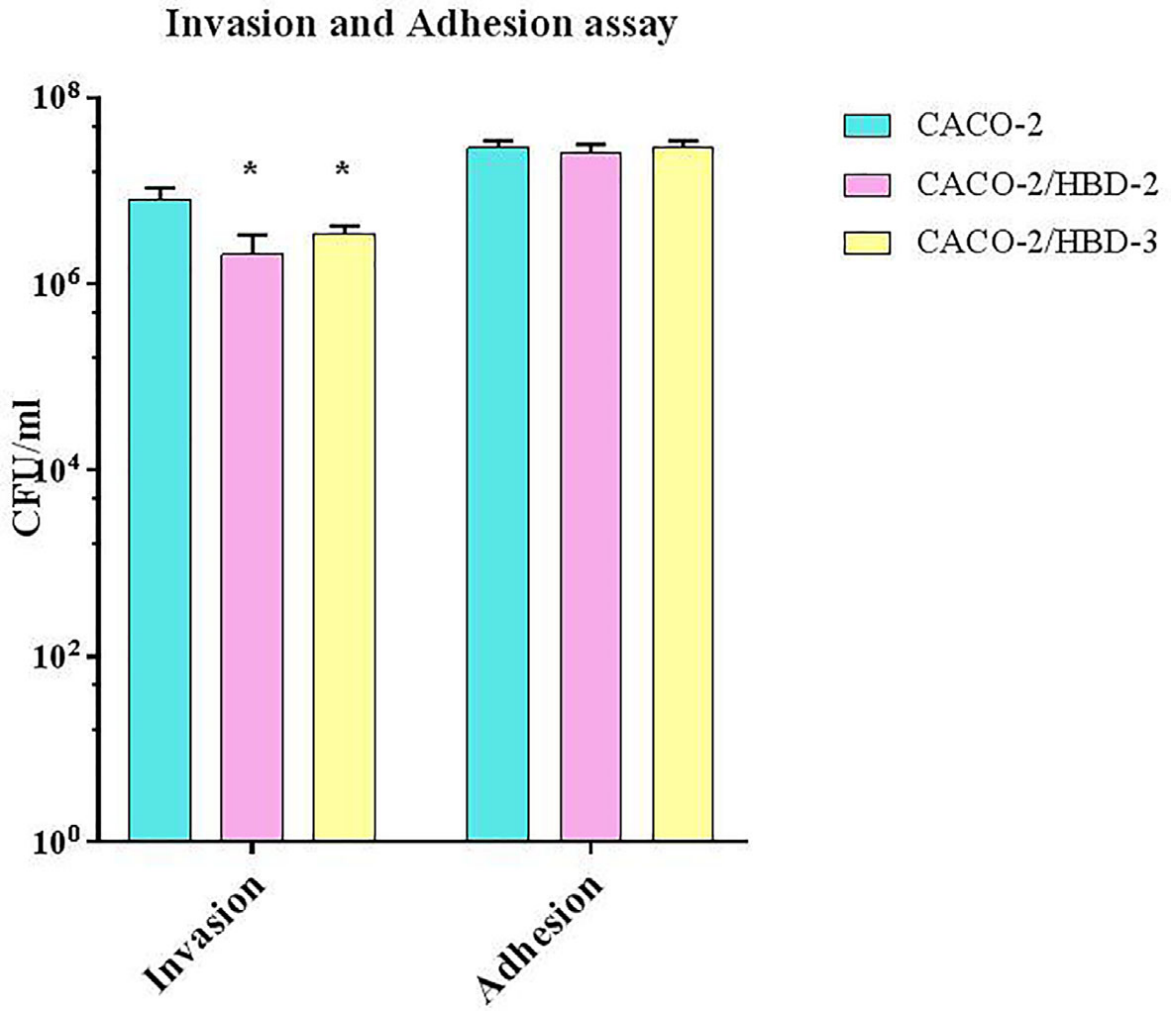
*C. albicans* invasion assays. Number of bacteria associated with Caco-2, Caco-2/HBD-2 and Caco-2/HBD-3 cells was determined by host cell lysis, plating, and counting CFU/ml. Data are representative of three different experiments ± SD. Significant differences are indicated by *p < 0.05, **p < 0.01, ***p < 0.001.

The invasiveness assay was carried out on *C. albicans* to evaluate the ability of the antimicrobial peptides to interfere with this pathogenic mechanism. As shown in **Figure 3**, while the adhesive ability of *C. albicans* does not appear to be influenced by the presence of antimicrobial peptides (~3x 10^7^ CFU/ml for Caco-2, ~2.6 x 10^7^ for Caco-2/HBD-2 and ~ 3x 10^7^ for Caco-2/HBD-3, with an initial inoculum of ~7 x 10^8^ CFU/ml) his invasive power was significantly reduced in Caco-2/HBD-2 (~2x10^6^ CFU/ml) and Caco-2/HBD-3 (~3.5x10^6^ CFU/ml) cells with respect to untransfected cells (~8x10^6^ CFU/ml).

### *C. albicans* Invasion Through the Barrier

**Figure 4:**
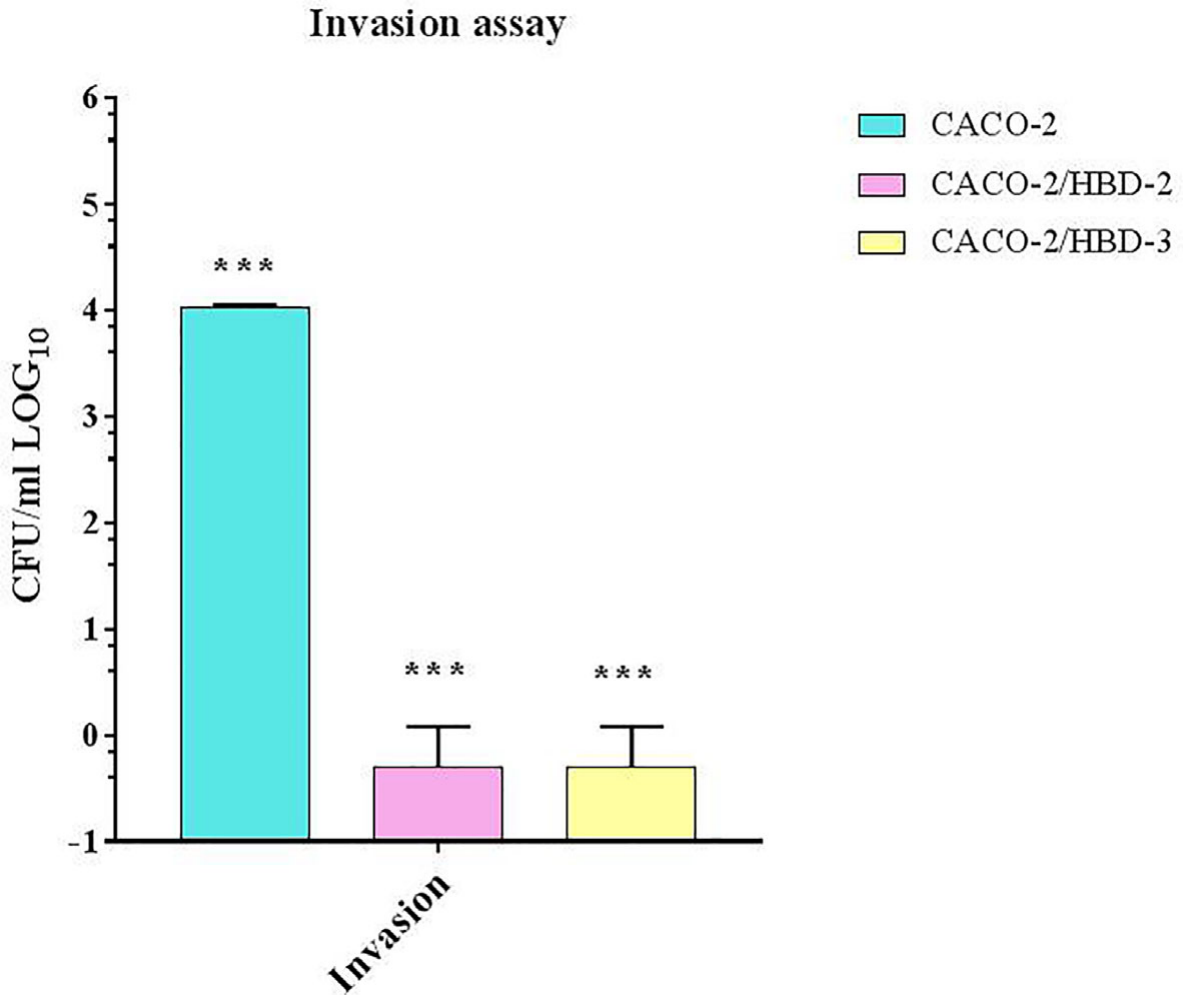
*C. albicans* invasion assays in Transwell^®^. Number of bacteria able to cross the barrier was determined by plating the yeast in the basolateral site, and counting CFU/ml. Data are representative of three different experiments ± SD. Significant differences are indicated by *p < 0.05, **p < 0.01, ***p < 0.001.

The invasion assay was conducted to confirm that the presence of antimicrobial peptides, by increasing the expression levels of the tight junctions proteins, strengthened the resistance of the intestinal barrier to prevent the passage of *C. albicans*. As shown in **Figure 4**, the results obtained show that, while in the untransfected cells *C. albicans* is able, after 4 h of infection, to cross the barrier with an invasion efficiency of 10^4^ CFUs/ml, in transfected cells the invasiveness degree is null.

### Cell Damage Induced by *C. albicans* Infection

**Figure 5:**
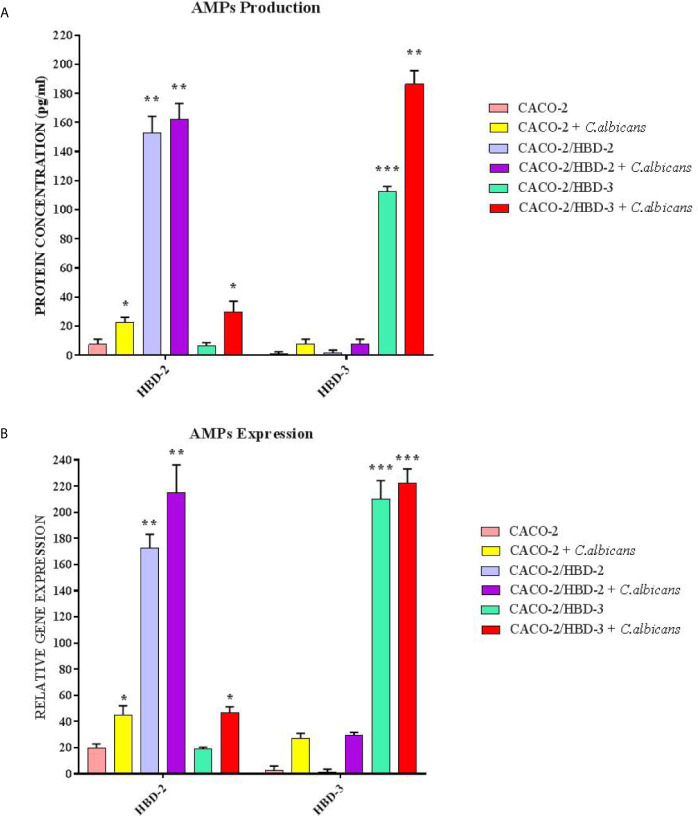
ELISA**(A)** and Real-Time PCR **(B)** expression in Caco-2, Caco-2/HBD-2 and Caco-2/HBD-3 cells infected with *C. albicans*. Data are expressed as relative protein concentration **(A)** and mRNAs expression **(B)** in each group, and are representative of three different experiments ± SD. Significant differences are indicated by *p < 0.05, **p < 0.01, ***p < 0.001.

**Figure 6:**
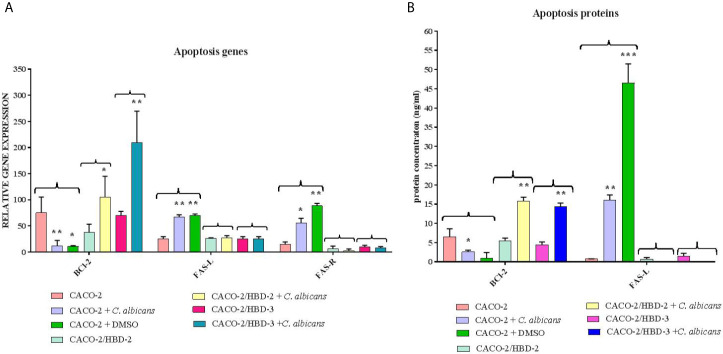
Real-Time PCR **(A)** and ELISA **(B)** results show the effects of HBD-2 and HBD-3 on expression of apoptotic genes and proteins infected with *C*. *albicans*. One well of untransfected cells was treated with DMSO as a positive cytotoxic control. Data are expressed as relative mRNAs expression **(A)** and protein concentration **(B)** in each group, and are representative of three different experiments ± SD. Significant differences are indicated by *p < 0.05, **p < 0.01, ***p < 0.001.

Caco-2, Caco-2/HBD-2 and Caco-2/HBD-3 cells were infected for 6 and 24 hours with *C. albicans* in order to verify the persistence of overexpression of AMPs and the activation of the apoptotic pathway. As positive cytotoxicity control, a well of each cell line was treated with 15% dimethyl sulfoxide (DMSO). The results obtained (**Figure 5**) first of all show that the expression of AMPs following infection with *C. albicans* increases in Caco-2 (especially HBD-2), and remains constantly very high in Caco-2/HBD-2 and Caco-2/HBD -3. Furthermore, as shown in **Figure 6A**, it can be seen that after 6 hours of infection in untransfected cells, an apoptotic process probably starts with the upregulation of the pro-apoptotic genes *FAS-L* and *FAS-R* and there is a downregulation of the anti-apoptotic gene *BCl-2*. This data is confirmed with the results obtained after 24 hours of infection, by ELISA assay (**Figure 6B**), where the upregulation of apoptotic pathway is confirmed also at the protein level, and by viability test conducted with the Alamar blue method (**Table 2**), when only 20% of the infected cells are metabolically active. The opposite trend is shown by the transfected cells, which shows upregulation of *BCl-2*, downregulation of *FAS-L* and *FAS-R*, and an almost intact percentage of active cells.

**Table 2:** % of ABred in Caco-2, Caco-2/HBD-2 and Caco-2/HBD-3 cells infected for 24 hours with *C. albicans*.

SAMPLE	%AB_RED_
**CACO-2+ *C.albicans***	20 ± 5
**CACO-2/HBD-2+ *C.albicans***	95 ± 5
**CACO-2/HBD-3+ *C.albicans***	92 ± 5
**CACO-2+DMSO**	3 ± 5
**CACO-2/HBD-2+DMSO**	2 ± 5
**CACO-2/HBD-3+ DMSO**	3 ± 5

## Discussion

The integrity and functionality of the intestinal barrier, which ensures the maintenance of homeostasis and the protection of the host from the attack of unwanted pathogens, are regulated by the strength of the binding between enterocytes and by a continuous communication with the resident microbiota and the immune system (Robinson et al., 2015; Vitale et al., 2016; Allaire et al., 2018).

Due to the similarity between Caco-2 monolayers and human intestinal epithelial cell barrier in morphology with the same cell polarity and TJs structure (Sambuy et al., 2005), we created a clone of Caco-2 cells stably transfected with antimicrobial peptides HBD-2 and HBD-3 to determine if their protective role in the intestinal barrier is associated with the expression of TJ proteins.

It is in fact known that dysfunctions of the barrier and diseases due to its reduced integrity are often associated with a decreased expression of TJs (Fukui, 2016; Oshima and Miwa, 2016; Sturgeon and Fasano, 2016; Andersen et al., 2017; Capaldo et al., 2017; Muñoz et al., 2019; Tsukita et al., 2019) and with an aberrant synthesis of AMPs by intestinal epithelial cells (Gácser et al., 2014; Robinson et al., 2015; Hancock et al., 2016).

In the first part of our experiments, we evaluated the effect of antimicrobial peptides on cell differentiation and on the integrity of the barrier. For this purpose, transfected and untransfected Caco-2 cells were cultured in Transwell^®^ for 21 days until complete differentiation was achieved. During this period, TEER was measured at regular time intervals and, at the end, expression of TJs and Mucin-2 was evaluated both by Real-Time PCR and by Western Blot and ELISA assay, respectively. The results obtained show that the transfected cells show a higher level of differentiation and integrity of the mucosa. In fact, the TEER is constantly higher than the untransfected cells for the whole period of differentiation, and the barrier proteins are much more prominently expressed in the presence of antimicrobial peptides. Indeed, we see that HBD-2 induces the expression of Mucin-2 and Occludin, while HBD-3 strongly increases the expression of Occludin, Zonulin and Claudin.

These data indicate that antimicrobial peptides strengthen the integrity of the barrier, thus helping to protect the host from alterations in intestinal permeability and subsequent microbial translocation.

Dysbiosis can cause alterations in the functioning of the intestinal barrier, which leads to an increase in the permeability of the mucosa with consequent release of proinflammatory factors and promotion of translocation of opportunistic pathogens (Oshima and Miwa, 2016; Nagpal and Yadav, 2017; Okumura and Takeda, 2018), including *C. albicans* (Basmaciyan et al., 2019; Bertolini et al., 2019), leading to localized or disseminated infections.

It has been widely proven that *C. albicans* is able to induce the production of HBD-2 in human intestinal epithelial cells (Cobo et al., 2015), however, the concentrations of antimicrobial peptide produced following contact with the fungus are not sufficient to guarantee the protection of the host from the onset of infection. In the second part of our experiments, we investigated the role of HBD-2 and HBD-3 in the pathogenesis of *C. albicans* infection, and in particular during the invasion within enterocytes and between the barrier, in order to evaluate whether the exogenous intake of peptides, in concentrations higher than those physiologically produced, could be of therapeutic interest in the treatment of infections caused by *C. albicans.*

The invasiveness tests conducted on transfected and untransfected Caco-2 cells show that the presence of antimicrobial peptides not only significantly inhibits the internalization of *C. albicans*, but also blocks its invasion through the monolayer, therefore through the barrier. Both of these effects can be due to the strengthening of the integrity of the barrier, confirmed by the increase in the expression of TJs and in TEER values. It is in fact known that barrier proteins not only regulate the passage of *C. albicans* between cells, but also its endocytosis (Allert et al., 2018).

The degree of metabolic activity was also measured, and the expression of apoptotic genes after infection with *C. albicans* was evaluated.

In particular, we analyzed the expression of BCl-2, FAS-L and FAS-R, both at molecular and protein level. BCl-2 is an intracellular protein with anti-apoptotic activity, induced by different stimuli in different cell types (Reed, 1994). FAS-L is a transmembrane protein, belonging, together with its FAS-R receptor, to the Tumor Necrosis factor -α (TNF-α) and its receptor families (Cosman, 1994). Activation of FAS-R by Fas-L induces the initiation of the apoptotic process (Cosman, 1994). It has been shown that the alteration of the balance between the expression of BCl-2 and FAS-L/FAS-R is involved in the pathology of various pathologies, such as autoimmune diseases, HIV, neurodegenerative disorders, cancer (Solary et al., 1996).

The results obtained show that the cells transfected with HBD-2 and HBD-3 show, after 6 hours of infection, a lower level of expression of the pro-apoptotic genes and a higher level of the antiapoptotic gene and, consequently, after 24 hours of infection, a significantly lower cell damage rate than untransfected cells.

It is already known that antimicrobial peptides possess antiapoptotic activity (Steubesand et al., 2009; Nagaoka et al., 2012), and in our experimental model, we have shown that their overexpression in the pathogenesis of *C. albicans* infections enhances this antiapoptotic power, as it also strengthens the integrity of the intestinal barrier, HBD -2 and HBD-3 reduce the invasive power of *Candida* and the cellular damage associated with it, responsible for the activation of the apoptotic pathway. An increasingly in-depth study of the interaction between antimicrobial peptides and microbiota will allow us to better clarify the mechanisms underlying their role in defense of host against intestinal pathogens and may one day turn the idea of therapies based on the use of AMPs into reality.

Currently, a valid strategy appears to be the integration of AMPs through the intake of nutraceuticals, probiotics and/or functional foods or able to induce AMPs synthesis in the gastrointestinal tract. This may have the potential to reduce or replace the use of antibiotics in animal/food production.

An even more ambitious prospect would be the translational use of AMPs in the clinical setting and in other contexts; however, to achieve such high-level milestones there is a need to improve understanding of the functioning of AMPs in their natural contexts and to understand how their evolutionary history can predict their future usefulness.

## Data Availability Statement

The raw data supporting the conclusions of this article will be made available by the authors, without undue reservation.

## Author Contributions

AF and GD designed the study. VS, MD, and BP oversaw the laboratory procedures. AF and BP wrote the manuscript. AF and GD supervised and validated the original draft. All authors contributed to the article and approved the submitted version.

## Conflict of Interest

The authors declare that the research was conducted in the absence of any commercial or financial relationships that could be construed as a potential conflict of interest.
