# Supplementary figures and images for: Antimicrobial Peptides Human Beta-Defensin-2 and -3 Protect the Gut During Candida albicans Infections Enhancing the Intestinal Barrier Integrity: In Vitro Study
Source: Front Cell Infect Microbiol. 2021 Jun 10;11:666900. doi: 10.3389/fcimb.2021.666900 (PMC8223513; doi:10.3389/fcimb.2021.666900)

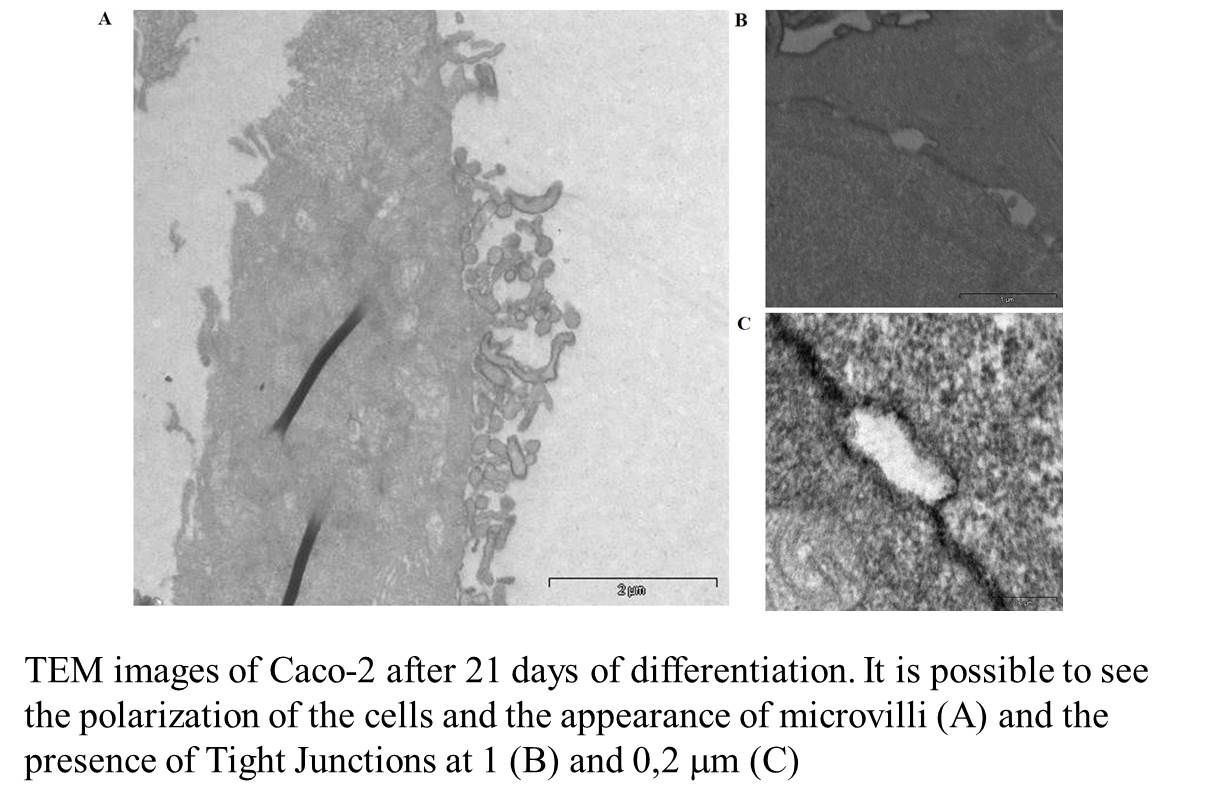

Supplement: Supplementary file 1 [file Image_1.jpeg]
